# A Systems Biology-Based Investigation into the Pharmacological Mechanisms of Wu Tou Tang Acting on Rheumatoid Arthritis by Integrating Network Analysis

**DOI:** 10.1155/2013/548498

**Published:** 2013-03-28

**Authors:** Yanqiong Zhang, Danhua Wang, Shufang Tan, Haiyu Xu, Chunfang Liu, Na Lin

**Affiliations:** Institute of Chinese Materia Medica, China Academy of Chinese Medical Sciences, No. 16, Nanxiaojie, Dongzhimennei, Beijing 100700, China

## Abstract

*Aim*. To investigate pharmacological mechanisms of Wu Tou Tang acting on rheumatoid arthritis (RA) by integrating network analysis at a system level. *Methods and Results*. Drug similarity search tool in Therapeutic Targets Database was used to screen 153 drugs with similar structures to compositive compounds of each ingredient in Wu Tou Tang and to identify 56 known targets of these similar drugs as predicted molecules which Wu Tou Tang affects. The recall, precision, accuracy, and F1-score, which were calculated to evaluate the performance of this method, were, respectively, 0.98, 0.61, 59.67%, and 0.76. Then, the predicted effector molecules of Wu Tou Tang were significantly enriched in neuroactive ligand-receptor interaction and calcium signaling pathway. Next, the importance of these predicted effector molecules was evaluated by analyzing their network topological features, such as degree, betweenness, and *k*-coreness. We further elucidated the biological significance of nine major candidate effector molecules of Wu Tou Tang for RA therapy and validated their associations with compositive compounds in Wu Tou Tang by the molecular docking simulation. *Conclusion*. Our data suggest the potential pharmacological mechanisms of Wu Tou Tang acting on RA by combining the strategies of systems biology and network pharmacology.

## 1. Introduction

Rheumatoid arthritis (RA) is a systemic autoimmune disease characterized by the presence of inflammatory synovitis, the predominance of proerosive mediators, and the progressive destruction of cartilage and bone [1]. Traditional Chinese Medicine (TCM) has been extensively used for centuries in the treatment of arthritic diseases. On the concept of TCM, RA is categorized as “arthromyodynia” (Bi Zheng, Bi syndrome, or blockage syndrome) [2]. Various TCM-based herbal formulas and the extracts or the ingredients of herbs, such as Wu Tou Tang, Guizhi Shaoyao Zhi Mu Tang, Du Huo Ji Sheng Tang, Fangji Huangqi Tang, and extracts of the herb Tripterygium Wilfordii Hook f. (TWHF) have been demonstrated to be effective for relieving the severity of RA [3–5]. The use of a combination of multiple herbs in TCM formulas is designed to exploit the additive or synergistic activities of individual herbs, as well as to balance or neutralize the toxic effects of certain herbal components by others in the mixture [6]. It is of great significance to screen effective ingredients from natural herbs and investigate their therapeutic mechanisms.

Wu Tou Tang as a classic TCM formula from Chinese medical sage Zhang Zhongjing is prepared from a basic formula of five Chinese herbs, including Radix Aconiti (Wu Tou), Herba Ephedrae (Ma Huang), Radix Astragali (Huang Qi), Raidix Paeoniae Alba (Bai Shao), and Radix Glycytthizae (Gan Cao). It is widely produced in China in accordance with the China Pharmacopoeia standard of quality control and is extensively used for the treatment of RA, hemicranias, and constitutional hypotension [7]. In TCM theory, multiple agents contained in one formula must work synergistically. With regard to Wu Tou Tang, Radix Aconiti is the primary component and is believed to be effective in treating rheumatic arthritis and RA; Herba Ephedrae serves as the ministerial component to intensify the analgesic function of Radix Aconiti; Radix Astragali acts as the adjunctive component to invigorate qi (vital energy), strengthen the body, and reinforce the effect of Radix Aconiti and Herba Ephedrae; Raidix Paeoniae Alba and Radix Glycytthizae are both messenger drugs which can either focus the actions of the formula on a certain area of the body or harmonize and integrate the actions of the other ingredients of the formula [8, 9]. There have been a large number of studies which were carried out to investigate in active monomers among ingredients of Wu Tou Tang and made great progresses. For example, Aconitine (monomer of Radix Aconiti) is found to greatly lighten the hyperalgesia of the rat adjuvant arthritis [10]; Ephedrine (monomer of Herba Ephedrae) is an alkaloid that functions as a decongestant, an antitussive, a central nervous system (CNS) stimulant, and an appetite suppressant [11]. However, monomer pharmacological effects cannot present overall efficacy of the whole formula. There is an urgent need for investigations involving all the compounds of Wu Tou Tang.

TCM, characterized by the use of herbal formula (Fu-Fang), is multicomponent and multitarget agents, essentially achieving the therapeutic effectiveness through collectively modulating the molecular network of the body system using its active ingredients. Which the development in high-throughput detection methods, many researchers have investigated the multitarget and synergetic actions of multicomponent in TCM formula at a molecular level. However, these studies have confronted several great challenges as following. First, it is very labor-intensive, time-consuming and costly to isolate and identify chemical constituents with desirable pharmacological effects, because most medicinal herbs may contain tens of thousands of constituents; then, it is very difficult to investigate its pharmacological and toxicological effects, because a certain component in TCM formula may act on multiple biological targets; finally, there may be a complex and highly dynamic ingredient-ingredient interaction network underlying the overall clinical effects because TCM formulas are administrated as an integrated prescription for treating diseases traditionally. In this context, it is necessary to develop a novel method which can understand the biological processes of the interactions among genes, proteins, and environmental factors at a system level in order to discover the molecular mechanisms related to the therapeutic efficacy of TCM. Network pharmacology, a novel research field which elucidates the underlying mechanisms of biological systems by analyzing various biological networks such as ingredient-ingredient, ingredient-target, target-target interaction networks, may have the potentials to address the relationship between multicomponents and drug synergistic effects [12, 13]. There are two kinds of approaches in network pharmacology. (1) Bottom-up: addition of well-known molecular drugs and observation of synergistic effects; (2) top-down: reduction of more general formula to its minimal elements that keep its beneficial properties [14, 15]. As TCM formula is considered to be an empirical system of multicomponent therapeutics which potentially meets the demands of treating a number of complex diseases in an integrated manner, the methodologies of network pharmacology are suitable for pursuing a priori knowledge about the combination rules embedded in TCM [16]. Therefore, we here intend to investigate the pharmacological mechanisms of Wu Tou Tang acting on RA by integrating network analysis. 

## 2. Materials and Methods

**Figure 1:**
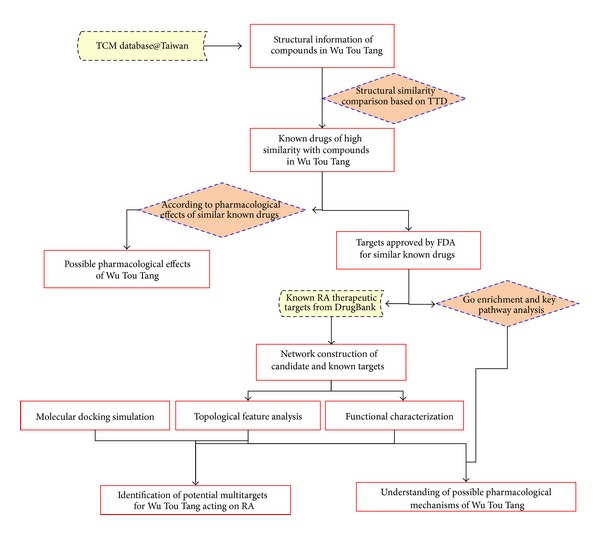
A schematic diagram of this systems biology-based investigation into the pharmacological mechanisms of Wu Tou Tang acting on rheumatoid arthritis by integrating multitarget identification and network analysis.

The technical strategy of this study was shown in Figure 1.

### 2.1. Data Preparation

#### 2.1.1. Structural Information of the Compositive Compounds of Each Ingredient in Wu Tou Tang

Structural information (*.mol or *.sdf files) of the compositive compounds of each ingredient in Wu Tou Tang was obtained from TCM Database@Taiwan [17] (http://tcm.cmu.edu.tw/, Updated in 2012-06-28), which is currently the largest noncommercial TCM database worldwide. TCM Database@Taiwan is based on information collected from Chinese medical texts and scientific publications and contains more than 20,000 pure compounds isolated from 453 TCM ingredients. In total, we collected the structural information of 17 compounds for Radix Aconiti, 29 compounds for Herba Ephedrae, 22 compounds for Radix Astragali, 15 compounds for Raidix Paeoniae Alba and 82 compounds for Radix Glycytthizae. 

#### 2.1.2. Known Therapeutic Targets Approved by FDA for the Treatment of RA

Known therapeutic targets were obtained from DrugBank database [18] (http://www.drugbank.ca/, version: 3.0). We only used those drug-target interactions whose drugs are FDA approved for the treatment of RA and whose targets are human genes/proteins. In total, we obtained 58 known therapeutic targets. The detailed information on these known therapeutic targets is described in Supplementary Table S1 in Supplementary Material available online at http://dx.doi.org/10.1155/2013/548498.

#### 2.1.3. Protein-Protein Interaction (PPI) Data

PPI data were imported from eight existing PPI databases including Human Annotated and Predicted Protein Interaction Database (HAPPI) [19], Reactome [20], Online Predicted Human Interaction Database (OPHID) [21], IntAct [22], Human Protein Reference Database (HPRD) [23], Molecular Interaction Database (MINT) [24], Database of Interacting Proteins (DIP) [25], and PDZBase [26]. The detailed information on these PPI databases is described in Supplementary Table S2. In total, we obtained 6713 interactions between 3231 proteins.

### 2.2. Pharmacological Mechanism Analysis

#### 2.2.1. Screening of Similar Drugs and Prediction of Effector Molecules for Wu Tou Tang

We used drug similarity search tool in Therapeutic Targets Database [27] (TTD, http://xin.cz3.nus.edu.sg/group/cjttd/ttd.asp, Version 4.3.02 release on Aug 25th 2011) to screen similar drugs of Wu Tou Tang through the structural similarity comparison. TTD provides comprehensive information about efficient targets and the corresponding approved, clinical trial, and investigative drugs. All information provided in TTD is fully referenced. We only selected the drugs with high similar score (>0.85, similar ~ very similar) in the comparison with the structures of compositive compounds of each ingredient in Wu Tou Tang. The therapeutic targets of these similar drugs were also collected as predicted effector molecules of Wu Tou Tang. In total, we obtained 153 similar drugs and 56 predicted effector molecules of Wu Tou Tang. The detailed information on these similar drugs and predicted effector molecules is described in Supplementary Table S3.

In order to evaluate the performance of this prediction method, 59 FDA-approved drugs and their known targets were randomly collected from TTD (http://bidd.nus.edu.sg/group/cjttd/TTD_Download.asp). The detailed information on these FDA-approved drugs and their known targets is described in Supplementary Table S4). As a certain drug may have or be predicted to have multiple targets, we used a multilabel evaluation measure which can rate predictions as “half-right” when only a portion of the correct labels were recovered or more labels than the correct ones were predicted. The overall accuracy (ACC) which is the percentage of correctly predicted instances, the recall (REC), the precision (PRE), and the average *F*1-score (*F*1) which is the harmonic mean of REC and PRE were calculated according to the previous study [28]. Let *D* denote a dataset with *n* instances. In addition, let *Y*_*i*_ and *Z*_*i*_ be the set of correct labels and the set of predicted labels of instance *i* ∈ *D*, respectively. Consequently, we can define the ACC, REC, PRE, and *F*1 for label *k* as follows:

(*f*1) $$\begin{array}{c} \text{ACC}=\sum_{\left(i/i\in D\right)}\frac{\left|Y_{i}\cap Z_{i}\right|}{\left|Y_{i}\cup Z_{i}\right|}, \end{array}$$

(*f*2) $$\begin{array}{c} \text{RE}{\text{C}}_{k}=\sum_{\left(i/i\in D\&k\in Y_{i}\right)}\frac{\left|Y_{i}\cap Z_{i}\right|}{\left|Y_{i}\right|}, \end{array}$$

(*f*3) $$\begin{array}{c} \text{PR}{\text{E}}_{k}=\sum_{\left(i/i\in D\&k\in Z_{i}\right)}\frac{\left|Y_{i}\cap Z_{i}\right|}{\left|Z_{i}\right|}, \end{array}$$

(*f*4) $$\begin{array}{c} FI=\frac{2\left(\text{REC}\right)\left(\text{PRE}\right)}{\text{REC}+\text{PRE}}. \end{array}$$

#### 2.2.2. Gene Ontology (GO) and Pathway Enrichment Analysis for Candidate Effector Molecules of Wu Tou Tang

We used Database for Annotation, Visualization, and Integrated Discovery [29] (DAVID, http://david.abcc.ncifcrf.gov/home.jsp, version 6.7) for GO enrichment analysis. DAVID now provides a comprehensive set of functional annotation tools for investigators to understand biological meaning behind a large list of genes. We also performed pathway enrichment analysis using pathway data obtained from the FTP service of KEGG [30] (Kyoto Encyclopedia of Genes and Genomes, http://www.genome.jp/kegg/, last updated: October 16, 2012). The KEGG pathway section is a collection of manually constructed pathway maps representing information on molecular interaction and reaction networks.

### 2.3. Network Construction

We first constructed a PPI network for known targets of RA and candidate effector molecules of Wu Tou Tang based on their PPI data obtained from eight existing PPI databases as mentioned above. Then, we applied Navigator software (Version 2.2.1) to visualize the PPI network.

### 2.4. Defining Features Set

For each node *i* in the above PPI network, we defined three measures for assessing its topological property. (1) “Degree” is defined as the number of links to node *i*; (2) “betweenness” is defined as the number of edges running through node *i*. Both degree and betweenness centrality can measure a protein's topological importance in the network. The larger a protein's degree/betweenness centrality is, the more important the protein is in the PPI network [31]. (3) *K*-core analysis is an iterative process in which the nodes are removed from the networks in order of least connected [32]. The core of maximum order is defined as the main core or the highest *k*-core of the network. A *k*-core subnetwork of the original network can be generated by recursively deleting vertices from the network whose degree is less than *k*. This results in a series of subnetworks that gradually reveal the globally central region of the original network. On this basis, “*K* value” is used to measure the centrality of node *i*.

### 2.5. Molecular Docking Simulation

eHiTS software [33] (Version 4.5, SimBioSys Inc. Canada) was used to validate the associations of candidate effector molecules with compositive compounds in Wu Tou Tang. All the protein structures were obtained from RCSB protein data bank [34] (http://www.pdb.org/, been updated in 2012-11-06) and have carefully checked for their resolutions. The 3D structures (*.mol files) of the compositive compounds of each ingredient in Wu Tou Tang were obtained from TCM Database@Taiwan [17] (http://tcm.cmu.edu.tw/, Updated in 2012-06-28). A docking score calculated by the customizable scoring function of eHiTS, which combines novel terms (based on local surface point contact evaluation) with traditional empirical and statistical approaches [33], was used to measure the binding efficiency of each effector molecule to the corresponding compound. For candidate effector molecules, when the docking score was higher than the median value, these proteins were identified be able to bind their corresponding compounds with strong binding efficiency.

## 3. Results and Discussion

### 3.1. Identification of the Underlying Pharmacological Mechanisms of Wu Tou Tang

In order to demonstrate the reliability of our prediction system, we firstly evaluated its performance by the multilabel measure. As the result of the independent set test, the recall value of our prediction system was 0.98, indicating that it could screen the effector molecules of drugs correctly; however, its precision (0.61) and overall accuracy (59.67%) were slightly low because of several false positive prediction results. The *F*1 score is a measure of a test's accuracy. It considers both the precision and the recall of the test to compute the score. The *F*1 score can be interpreted as a weighted average of the precision and recall, where an *F*1 score reaches its best value at 1 and worst score at 0. It is better suited than the overall accuracy, especially for unbalanced datasets, because the overall accuracy often biases towards an overrepresented class [28]. Our data have shown that the *F*1 score of our prediction system was 0.76. 

**Figure 2:**
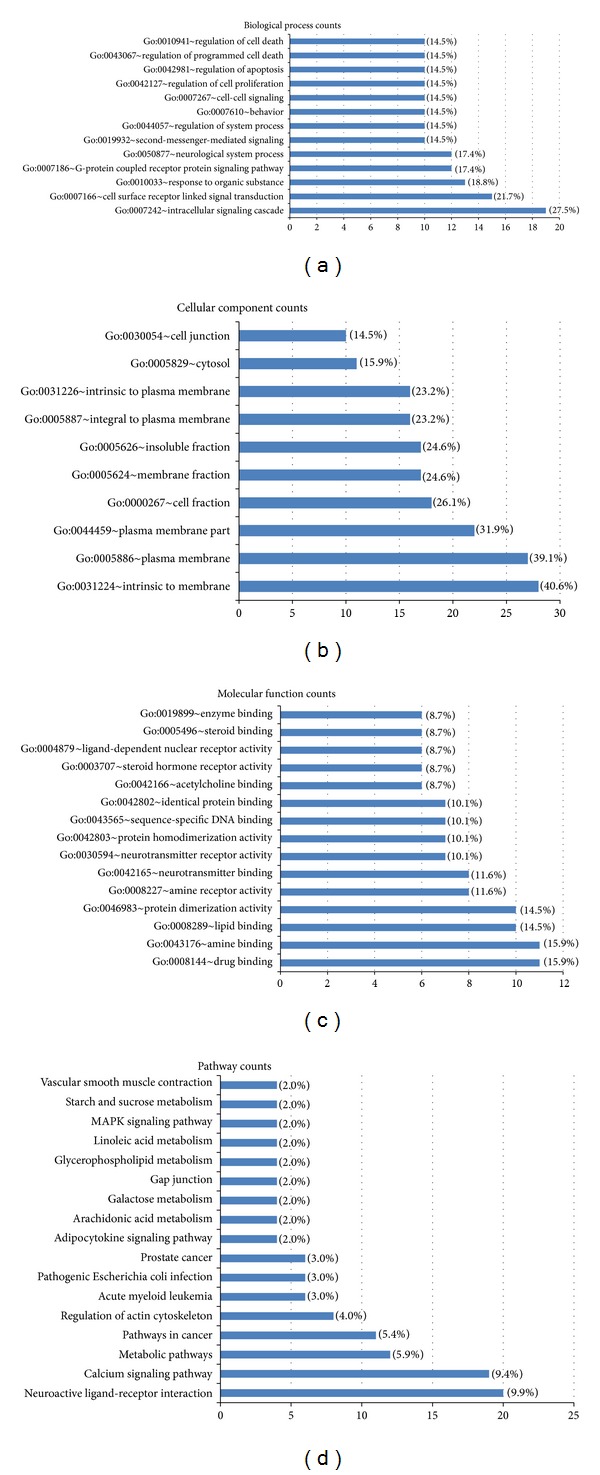
Enriched Gene Ontology terms for biological processes (a), for cellular components (b), and for molecular functions (c) and KEGG pathways (d) on the candidate effector molecules of Wu Tou Tang.

Using this prediction system, 153 similar drugs of compositive compounds (data shown in Supplementary Table S3) and 56 candidate effector molecules (data shown in Supplementary Table S3) for Wu Tou Tang were screened to reveal their underlying molecular mechanisms. According to the therapeutic effects of these similar drugs, we found that all five ingredients in Wu Tou Tang could act as anti-inflammatory, antibacterial, and hypoglycemic agents. Especially, both Radix Aconiti and Herba Ephedrae could be used as analgesics and corticosteroids; Radix Astragali had deintoxication; both Raidix Paeoniae Alba and Radix Glycytthizae could function as analgesics, immune regulator, and dietary supplement. In addition, we researched the functional distribution of candidate effector molecules of Wu Tou Tang by GO enrichment analysis. The GO annotation system uses a controlled and hierarchical vocabulary to assign function to genes or gene products in any organism. It contains three independent categories: biological processes, molecular function, and cellular components.  Figure 2 shows enriched GO terms of predicted effector molecules of Wu Tou Tang. The top three significantly enriched GO biological processes of them include intracellular signaling cascade, cell surface receptor linked signal transduction, and response to organic substance (Figure 2(a)); most of these predicted effector molecules are localized on the cellular membrane (Figure 2(b)) and function as binding components (Figure 2(c)). These annotations are all related with the processes of different molecular signal transmissions, indicating that Wu Tou Tang may intervene in these pathological progresses.

Pathway information is important for understanding gene and protein function. Therefore, we analyzed the enriched KEGG biological pathways among these predicted effector molecules of Wu Tou Tang. As shown in Figure 2(d), the most associated pathway was neuroactive ligand-receptor interaction, which had 20 (20/56, 35.71%) predicted effector molecules associated with it. The second-most frequent associations were calcium signaling pathway, followed by metabolic pathways, pathways in cancer, regulation of actin cytoskeleton, pathogenic Escherichia coli infection, and so on.

**Figure 3:**
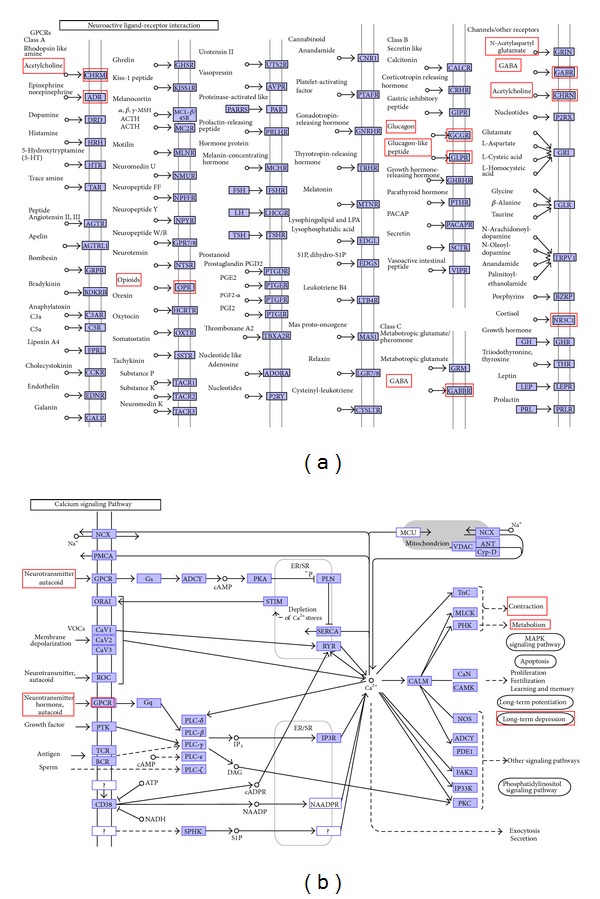
Effects of active compounds in Wu Tou Tang on neuroactive ligand-receptor interaction (a) and calcium signaling pathway (b) by their candidate effector molecules marked with red panes. This plot is modified from KEGG pathway map.

Among these pathways, the importance of neuroactive ligand-receptor interaction in the development and progress of RA has been reported. In TCM theory, patients suffering from RA can be categorized into Cold-ZHENG-related RA who are treated by the cold-warming herbal formulas and Hot-ZHENG-related RA who are treated by the hot-cooling herbal formulas [35]. Li et al. [36] found that genes shared by both Cold-ZHENG and Hot-ZHENG are significantly enriched in the pathway of neuroactive ligand-receptor interaction. In the present study, we mapped the predicted effector molecules of Wu Tou Tang onto KEGG pathways.  Figure 3(a) shows the effects of the active compounds in Wu Tou Tang on the system of neuroactive ligand-receptor interaction. These active compounds act on different receptors so as to regulate the uptake and transport systems of neurotransmitters such as acetylcholine, norepinephrine and opioid, suggesting that Wu Tou Tang may block the reuptake of multiple neurotransmitters and stimulate the release of these neurotransmitters in a multitarget pattern. In addition, it has been demonstrated that calcium signaling pathway plays an important role in RA progression. Ca^2+^ signals are essential for diverse cellular functions including differentiation, effector function, and gene transcription in the immune system. Davies and Hallett [37] found that cytosolic Ca^2+^ signaling could trigger neutrophil responses in RA. Lu et al. [38] also indicated that calcium signaling pathway may be related with heat pattern of RA. As shown in Figure 3(b), the predicted effector molecules of Wu Tou Tang mapped in this pathway such as adrenergic receptor and opioid receptor are closely related to the progression of RA.

### 3.2. Importance of the Candidate Effector Molecules for Wu Tou Tang Acting on RA Therapy

**Figure 4:**
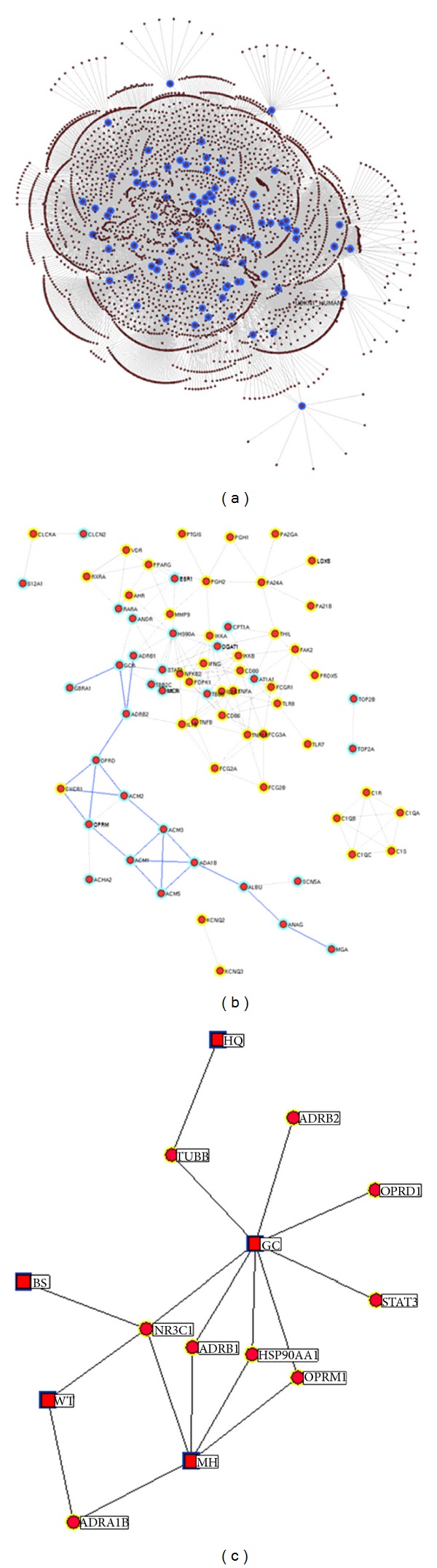
(a) The protein-protein interaction (PPI) network of known targets of rheumatoid arthritis (RA) and candidate effector molecules of Wu Tou Tang based on their PPI data. In total, there are 6713 interactions between 3231 proteins in the PPI network. (b) The PPI network of 129 hub proteins obtained from (a). Yellow nodes refer to known targets of RA, and green nodes refer to candidate effector molecules of Wu Tou Tang. Pathway with blue edges refer to neuroactive ligand-receptor interaction, which is the most associated pathway of candidate effector molecules of Wu Tou Tang according to the pathway enrichment analysis. (c) The interaction network of five ingredients in Wu Tou Tang and nine major candidate effector molecules of Wu Tou Tang on RA therapy. Square nodes refer to ingredients in Wu Tou Tang, and circular nodes refer to major candidate effector molecules of Wu Tou Tang on RA therapy.

We constructed a PPI network for known targets of RA and candidate effector molecules of Wu Tou Tang based on their PPI data. In total, there are 6713 interactions between 3231 proteins (Figure 4(a)). According to the previous study of Li et al. [36], we identified a node as a hub protein if its degree is more than 2-fold of the median degree of all nodes in a network. As the results, there are 129 hub proteins, the interactions among which are shown in Figure 4(b).

Analysis on topological features may improve the identification of essential proteins in PPI networks. Based on the characteristic of biological network, nodes with higher degree and *K*-coreness are the center of network and their removal may disrupt a number of essential pathways to break network [32, 39]. In addition, some global topological features such as closeness/betweenness centrality have been put forward. Closeness/betweenness centrality correlates more closely with essentiality than degree, exposing critical nodes that usually belong to the group of scaffold proteins or proteins involved in crosstalk between signaling pathways [31]. Nodes with higher value of closeness/betweenness centrality can be identified as initial candidates for drug targets [40]. Thus, we calculated the degree, the betweenness, and the *K*-coreness of candidate effector molecules in order to demonstrate their importance in PPI network. The nodes with all the three feature values (“Degree”, “Betweenness” and “*K* value”) higher than their corresponding medians were identified as major candidate effector molecules of Wu Tou Tang acting on RA. As the result, nine proteins, ADRB1_HUMAN (official gene symbol, OGC: ADRB1), ADRB2_HUMAN (OGC: ADRB2), OPRM_HUMAN (OGC: OPRM1), OPRD_HUMAN (OGC: OPRD1), ADA1B_HUMAN (OGC: ADRA1B), HS90A_HUMAN (OGC: HSP90AA1), STAT3_HUMAN (OGC: STAT3), GCR_HUMAN (OGC: NR3C1), and TBB5_HUMAN (OGC: TUBB), were identified as major candidate effector molecules of Wu Tou Tang on RA therapy. The detailed information on these proteins and their corresponding compounds in Wu Tou Tang is described in Supplementary Table S5. As shown in Figure 4(c), NR3C1 was the common effector molecules of four ingredients in Wu Tou Tang, including Radix Aconiti, Herba Ephedrae, Raidix Paeoniae Alba, and Radix Glycytthizae; ADRA1B was the common effector molecules of Radix Aconiti and Herba Ephedrae; ADRB1, HSP90AA1, and OPRM1 were all the common effector molecules of Herba Ephedrae and Radix Glycytthizae; TUBB was the common effector molecules of Radix Glycytthizae and Radix Astragali. All these data indicate that the therapeutic effects of Wu Tou Tang on RA may be based on the synergistic interactions of different ingredients.

#### 3.2.1. Biological Interpretations of Major Candidate Effector Molecules

Among nine major candidate effector molecules of Wu Tou Tang on RA therapy, ADRB2, ADRA1B, HSP90AA1, STAT3, NR3C1, and TUBB have been demonstrated to be associated with RA progression. We would like to illustrate their biological significance in RA.

ADRB2, named as beta-2 adrenergic receptor, is a member of the group of G-protein-coupled receptors [41]. It is present on skeletal and cardiac muscle cells and on peripheral blood lymphocytes. ADRB2 may represent a link between the sympathetic nervous system and the immune system [42]. In RA patients, Baerwald et al. [43] detected the reduced number of ADRB2 on peripheral blood mononuclear cells, which may be associated with disease activity and defective suppressor cell functions. Pont-Kingdon et al. [44] also indicated that ADRB2 might be a factor affecting RA by impairing the control of the immune response. These involvements of ADRB2 in RA imply a potential importance of its genetic variation in this disease. Xu et al. [45] showed an association of ADRB2 SNPs with RA in a population from the northern part of Sweden. Malysheva et al. [46] further demonstrated a correlation between ADRB2 polymorphisms and RA in conjunction with human leukocyte antigen-DRB1 shared epitope. These findings suggest the associations between RA and variants in the gene encoding ADRB2.

ADRA1B, named as alpha-1B adrenergic receptor, mediates its action by association with G proteins that activate a phosphatidylinositol-calcium second messenger system [47]. Its effect is mediated by G(q) and G(11) proteins. Nuclear ADRA1A-ADRA1B hetero-oligomers regulate phenylephrine (PE)-stimulated ERK signaling in cardiac myocytes. Previous studies demonstrated that the expression of ADRA1B mRNA in PBMC during chronic inflammation in juvenile rheumatoid arthritis (JRA) may be associated with altered responses of the immune system to stress [48].

HSP90AA1, named as heat shock protein HSP 90-alpha (Hsp90*α*), belongs to the heat shock protein 90 family [49]. It is a highly conserved and abundant protein, constituting approximately 1% of the total intracellular protein. This protein is localized in cytoplasm and melanosome of human cells. In the cytoplasm, Hsp90*α* has more than 200 interacting proteins, and it commonly functions in concert with various cochaperones including Hsp70, Hsp40, Hop, Hip, and p23, which can form a complex and subsequently bind to the interacting proteins and assist in their folding or activation [50]. Functionally, Hsp90*α* promotes the maturation, structural maintenance, and proper regulation of specific target proteins involved for instance in cell cycle control and signal transduction. Accumulating studies have indicated the intracellular role of Hsp90*α* in tumorigenesis. In 2011, Sedlackova et al. [51] detected the HSP expression profile by real-time quantitative reverse transcription polymerase chain reaction in RA, osteoarthritis, and healthy controls. Their data showed the significantly increased Hsp90*α* mRNA level in RA synovial tissues. This upregulation together with the downregulation of Hsp70 and the elevated HspBP1/Hsp70 mRNA ratios can be used to differentiate between RA patients and healthy individuals through analysis of peripheral blood samples, suggesting that the differential expression of Hsp90*α* may be a promising diagnostic marker for RA patients.

STAT3, named as signal transducer and activator of transcription 3, belongs to the transcription factor STAT family and contains one SH2 domain [52]. Among seven known STAT proteins, STAT3 has been demonstrated to be active in synovial lining cells in adjuvant arthritis and RA and in freshly isolated RA SFs [53]. It is activated by a number of cytokines and growth factors expressed in RA synovitis, including IL-6, oncostatin M, EGF, and PDGF [54]. STAT3 is one of components in the Janus kinase (JAK)-STAT signal transduction pathway, which functionally regulates gene expression and various cellular processes, including cell activation, proliferation, and differentiation. In RA, this pathway plays a critical role in synovial membrane proliferation. Emerging experimental results demonstrate that JAK-STAT inhibitors may exhibit dramatic effects on RA in clinical trials [55].

NR3C1, named as glucocorticoid receptor, has a dual mode of action, as a transcription factor that binds to glucocorticoid response elements and as a modulator of other transcription factors [56]. It affects inflammatory responses, cellular proliferation, and differentiation in target tissues. Glucocorticoids are extensively used in the treatment of inflammatory bone diseases, such as RA. Rauch et al. [57] indicated that the anti-inflammatory selective glucocorticoid receptor modulator may preserve osteoblast differentiation.

TUBB, named as tubulin beta chain, belongs to the tubulin family. Tubulin is the major constituent of microtubules [58]. It binds two moles of GTP, one at an exchangeable site on the beta chain and one at a nonexchangeable site on the alpha chain. In 1992, Ramos-Ruiz et al. [59] found the decreased tubulin synthesis in synoviocytes from adjuvant-induced arthritic rats. By proteomic analysis, Kamada et al. [60] further found that the expression levels of tubulin protein in bone marrow-adherent cells were increased in osteoarthritis compared to RA.

#### 3.2.2. Validation by Molecular Docking Simulation

Molecular docking simulation, as one of structure-based methods, is an invaluable tool in drug discovery and design. Computational docking technique is flexible ligand docking, where the candidate ligands are fitted to the 3D structure of the target receptor with allowance for the conformational flexibility of the ligands [61]. Thus, it is of great importance to investigate ligand-protein interactions and elucidate binding mechanisms. eHiTS, one of the molecular docking softwares, systematically covers the part of the conformational and positional search space that avoids severe steric clashes, producing highly accurate docking poses at a speed practical for virtual high-throughput screening [33]. For these reasons, the molecular docking simulation was performed in this study to validate the associations of major candidate effector molecules with compositive compounds of Wu Tou Tang on RA therapy using eHiTs software. As a result, 26 pairs of compound-candidate effector molecules interactions were deleted either because their structural information was unavailable or because negative results were output from eHiTS. The positive docking results for other interactions were summarized in Supplementary Table S6. The median value of all docking scores was −4.41 kcal/mol, and there were 13 pairs of compound-candidate effector molecules interactions with strong binding free energy. Among these, GCR_HUMAN could effectively bind with five “candidate compounds” (Aconitine from Radix Aconiti, Methyl-7-epiganoderate from Herba Ephedrae, Paeoniflorin from Raidix Paeoniae Alba, Isoramanone from Radix Glycytthizae, and Aldohypaconitine from Radix Aconiti); ADRB1_HUMAN could also efficiently bind with five “candidate compounds” (Isogosferol from Radix Glycytthizae, Neohancoside A from Radix Glycytthizae, delta-Terpineol from Herba Ephedrae, Neoisopulegol from Radix Glycytthizae, and trans-beta-Terpineol from Herba Ephedrae); STAT3_HUMAN could effectively bind with two compounds in Radix Glycytthizae (Methyl glycyrrhetate and Glycyrrhetol); ADRB2_HUMAN could efficiently bind with one compound in Radix Glycytthizae (2-Methy1-1,3,6-tri). Especially, the binding free energy of the Aconitine-GCR_HUMAN (−8.81 kcal/mol), Methyl-7-epiganoderate-GCR_HUMAN (−8.27 kcal/mol), and Paeoniflorin-GCR_HUMAN interactions (−6.75 kcal/mol) were all higher than 1.5-fold of the median value of docking scores.

## 4. Conclusion

Different from western medicine, TCM is an independent system of theory, which treats the function and dysfunction of living organisms in a more holistic way. It is very difficult to understand the therapeutic mechanisms of TCM because of the complexity of the chemical components and their actions in vivo. Many studies have applied monomer in herbs to elucidate the pharmacological efficacy of the whole TCM formula. However, this method ignored the multitarget characteristic of the multicomponent TCM formula. At present, to develop an effective method for understanding the TCM system as a whole is still the “bottleneck” of modern TCM study. Currently, we combined the strategies of systems biology and network pharmacology to investigate the complicated multitarget mechanisms of Wu Tou Tang. Our main findings are (1) to develop a novel strategy which is used to investigate into the therapeutic mechanisms of Wu Tou Tang from chemical structures, genomic, proteomic, and pharmacological data in an integrated framework. (2) This strategy can pinpoint out the underlying pharmacological effects of the ingredients in Wu Tou Tang based on the synergistic interactions of the ingredients, targets, and pathways. The results indicate that Radix Aconiti shares the most common effector molecules with Herba Ephedrae, while less common effector molecules with Radix Glycytthizae and Raidix Paeoniae Alba. Moreover, it is important to note that there may be the most common effector molecules between Radix Glycytthizae and Herba Ephedrae. These findings suggest that five Chinese herbs in Wu Tou Tang together probably display synergistic actions and our network-based approach may facilitate to generate hypothesis to optimize and reformulate the herbal formula by elucidating the compatible mechanism of the complex prescription. (3) We also provide a list of candidate effector molecules for Wu Tou Tang; some of them backed experimental evidence reported in the literature and were validated by the molecular docking simulation. Although there are potentially interesting associations between these effector molecules and RA, cautious interpretation should be performed as our strategy is based on statistical analysis. Therefore, further experimental studies are required to test these hypotheses. Taken together, this study may support further assessments of clinical application of Wu Tou Tang, and enable further research on TCM formulas in a more timely and cost-effective manner.

## Supplementary Material

Table S1: describes the detailed information on known drugs and therapeutic targets for the treatment of rheumatoid arthritis. It includes drug name, drug accession number, drug group, target name, target UniprotID and target gene symbol.
Table S2: describes the detailed information on eight existing protein-protein interaction databases including source name, website and release number/last updated date.
Table S3: describes the detailed information on the similar drugs of compositive compounds of each ingredient in Wu Tou Tang and the predicted effector molecules of Wu Tou Tang.
Table S4: describes the detailed information on the FDA-approved drugs for the treatment of rheumatoid arthritis and their known targets.
Table S5: describes the detailed information on the major candidate effector molecules and their corresponding compounds in Wu Tou Tang including drug compounds, drug target UniprotID, drug target UniprotACC and drug target gene symbol.
Table S6: describes the detailed information on the positive docking results for compound-potential target interactions including drug compound, drug target UniprotID, drug target UniprotACC, drug target gene symbol, drug target PDB ID and Ehits score.
